# Changes in substance use, recovery, and quality of life during the initial phase of the COVID-19 pandemic

**DOI:** 10.1371/journal.pone.0300848

**Published:** 2024-05-22

**Authors:** Megayn E. Lewandowski, Colette N. Delawalla, Tarah J. Butcher, Brandon G. Oberlin

**Affiliations:** 1 Department of Psychiatry, Indiana University School of Medicine, Indianapolis, Indiana, United States of America; 2 Department of Psychology, Indiana University-Purdue University Indianapolis, Indianapolis, Indiana, United States of America; 3 Department of Psychological Science, Ball State University, Muncie, Indiana, United States of America; Jawaharlal Institute of Postgraduate Medical Education and Research, INDIA

## Abstract

**Background:**

The COVID-19 pandemic disrupted lives on a massive scale. While the pandemic appeared to worsen mental health outcomes broadly, its effects on alcohol/substance use and recovery are unclear. Many studies convolved the sociopolitical unrest beginning in May 2020 with the pandemic. We assessed pandemic-related changes in substance use, recovery involvement, and quality of life among US adults at two specified time periods that isolated pandemic effects from potentially confounding sociopolitical factors.

**Objectives:**

We tested the following hypotheses: the pandemic and consequent policies (1) increased use of alcohol and illicit substances in active users; (2) increased use of alcohol/substances among people in early recovery; (3) reduced participation in recovery activities among those in early recovery, and that (4) use amount and use events correlated with impulsivity in both groups and that (5) substance use and abstinence correlated with resilience.

**Methods:**

We recruited 1,685 participants through Amazon’s Mechanical Turk (MTurk). We assessed demographics, quality of life, alcohol/substance use, recovery activities, and measures of impulsivity and resilience at two time points, pre-pandemic and (early) during-pandemic. Only *n* = 45 (Active Users; males *n* = 32) and *n* = 34 (Recovery; males *n* = 20) passed data quality checks and were included in the primary analyses.

**Results:**

Among Active Users, weekly alcohol consumption and days spent using alcohol and illicit substances decreased during the pandemic. Resilience negatively correlated with pandemic-related substance use in early recovering participants. Significant reduction in the quality of life was coincident with a trend of lower recovery activity participation (31% decline) during the pandemic.

**Conclusions:**

The reduced alcohol/substance use and participation in recovery activities might be expected from conditions that promote social isolation. The high prevalence of low-quality data from MTurk cautions for careful use of online data sourcing.

## 1. Introduction

The COVID-19 pandemic inflicted tremendous loss of life (~6 million globally by May 2022; World Health Organization, https://covid19.who.int/) and enormous economic casualties (~$16 trillion in the first year in the United States alone; [1]). These costs, coinciding with extended periods of social isolation, demoralizing media messaging, and restricted social opportunities, created societal upheaval and psychological damage on a large scale [2]. Prior to the pandemic, binge drinking in the US was relatively common, with ~22% of US adults reporting at least one past-year binge drinking day [3]. Binge drinking, particularly at higher levels, is associated with adverse health and social outcomes, including automobile accidents while intoxicated, physical fights and injuries, arrests, and emergency room visits [4]. Additionally, in 2019, substance use among U.S. adults 18 years and older approximated 36 million [5], with about 8 million illicit substance-related emergency room visits. The degree of harm to those requiring medical care for alcohol and drug use was likely enhanced in early 2020 due to medical services prioritizing Covid-related complications over other medical concerns. Nonetheless, the overall effect of the pandemic on alcohol and drug use is not obvious (large meta-analyses; [6, 7])

The degree to which profoundly negative life events affect substance use and recovery is modulated by resources (external) and personality (internal) factors. Externally, low quality of life in the domains of personal goals and standards of living, and negative life events can exacerbate the harm from alcohol and drugs [8, 9]. These factors are even more prevalent in comorbid alcohol and substance use disorder (ASUD) populations [10, 11]. Conversely, one’s perceived quality of life is an important factor in coping with negative life events during early recovery from ASUDs [12]. Perceived quality of life is affected by major life events such as loss of employment, divorce, or injury/illness—all of which increase the risk of substance use [13]. The perceptual impact of life events is influenced by active substance use or ASUD recovery [14, 15], suggesting that the direct effects of substance use may dynamically interact with subjective factors. While numerous internal factors influence ASUD and recovery, two key factors include resilience and impulsivity. Resilience is the capacity to cope with stressors [16], and correlates with abstinence in individuals with ASUDs [17]. We expect resilience to predict better outcomes for addiction-related behaviors during a prolonged stressor, both for active substance use and recovery. In contrast, impulsivity confers risk for poor outcomes, and is characterized by a conglomerate of maladaptive behaviors characterized by inattention to future consequences. The three main components of classically-defined impulsivity are: a decreased sensitivity to negative consequences of behavior, a lack of foresight about how current decisions affect the future, and immediate or unplanned reactions to environmental stimuli before fully processing all of the information [18]. Impulsive traits and behavior, measured with self-report [19, 20] or behavioral tasks [21, 22], robustly correlate with ASUD and recovery outcomes [23–30].

The present study aimed to characterize changes in substance use related to the COVID-19 pandemic in participants actively using alcohol and/or illicit substances and participants in early recovery from ASUDs. Similar studies examining drinking and substance use patterns during the pandemic have produced mixed results [6, 7]. Importantly, many of these have averaged over a time period that included substantial sociopolitical upheaval. We assessed two specific 45-day periods to isolate pre- and during-COVID. The COVID-19 outbreak was declared a pandemic by the WHO on March 11^th^, 2020 (Center for Disease Control, https://www.cdc.gov/museum/timeline/covid19.html). The pre-COVID period was designated as January 17^th^, 2020, to March 1^st^, 2020. To avoid conflating the sociopolitical unrest beginning May 25^th^ with purely pandemic-related factors, the during-COVID period was designated as (April 1^st^, 2020, to May 15^th^, 2020). We hypothesized that, relative to pre-COVID, we would find (1) increased use of alcohol and illicit substances, including marijuana, during-COVID among the Active User group; (2) greater drug use during-COVID among the (early) Recovery group; (3) reduced recovery involvement during-COVID among the Recovery group; and (4) that higher impulsivity (behavioral and self-reported) would correlate with increased use in both groups (Active User and Recovery); and (5) that higher resilience will correlate with better use-related outcomes (reduced consumption of alcohol and illicit substance or sustained abstinence).

## 2. Methods

### 2.1. Participants

Seventy-nine participants were recruited through Amazon Mechanical Turk (MTurk), and put into two groups (Recovery, *n =* 34; Active User, *n =* 45) and analyzed separately (also see section 3.3 “Excluded Data”). Assessments were completed from August 7^th^ to August 16^th^, 2020. Eligibility requirements: United States residents, at least 18 years of age, and possessing a valid MTurk Worker account. Further inclusion criteria included: (1) the current use of illicit substances (including marijuana) or meeting the National Institute on Alcohol Abuse and Alcoholism guidelines for heavy drinking (consumption of more than seven alcoholic drinks per week for females or more than fourteen drinks per week for males; https://www.niaaa.nih.gov/alcohol-health/overview-alcohol-consumption/moderate-binge-drinking), OR (2) being in early recovery from an ASUD (e.g., currently in treatment, or actively participating in recovery activities, with continuous alcohol/drug abstinence no longer than 18 months), and (3) passing attention checks (see 2.2 Assessment), which included the successful completion of a control trial during the 5-trail delay discounting task. In addition, after rejecting most participant data (*n* = 1,606) for suspicious bot or non-US status (e.g., fillable text fields containing incoherent responses or non-English syntax), we further restricted inclusion by requiring (4) a Human Intelligence Task (HIT) of 90%. A HIT approval rating is a percentage calculated based on the number of tasks completed successfully and is MTurk’s metric of presumed quality. Only 5% of the Recovery and Active User participants met all inclusion criteria, leaving a final sample of 79 participants for data analysis. All assessments were completed on Qualtrics, with some assessments administered twice (once for the pre-COVID condition [January 17^th^, 2020, to March 1^st^, 2020] and once for the during-COVID condition [April 1^st^, 2020, to May 15^th^, 2020]). Participants were compensated $3 upon completing the survey and the 5-trial delay discounting task.

US-based internet access location was verified by cross-referencing the physical location of participants’ IP address registration with the self-reported zip code on Qualtrics. In addition, IP2Location services (https://www.ip2location.com/web-service/ip2location) were used to determine if participants were likely using a VPN or proxy service to mask their location. Finally, a subset (*n* = 113) of participants whose US location could not be verified using this method but met all other inclusion criteria were analyzed separately for generalizability of results (see Supporting Information). We presumed that these were real humans, but potentially non-US, so therefore provided a useful comparison.

This study was approved by the Indiana University at Indianapolis Human Research Protection Program Institutional Review Board. Once MTurk participants accessed the link to the survey the link provided a study information sheet prior to completing any study procedures (in lieu of a formal consent document). The study information sheet was one page in length and had a three-minute time-lock on it so the experimenters could be assured the participants had enough time to read the study information sheet and decide if they still wished to participate. Participation was voluntary, and participants could end their participation at any time.

### 2.2. Assessment

*Demographic* information included participants’ age, biological sex, race and ethnicity, total monthly income and disposable income, nicotine use, the highest level of education attained (bins: some high school [<11 years], high school diploma/GED [12 years], some college [14 years], bachelor’s degree [16 years], advanced degree [18 years]), academic major if a current part-time or full-time student, current occupation, zip code, household income during childhood (bins: <$10,000; $10,000-$24,000; $25,000-$49,000; $50,000-$74,000; $75,000-$99,000; $100,000-$200,000; >$200,000), and parent’s education and occupation when the participant was 18 years of age.

*Fidelity checks* assessed attention and identified potentially fraudulent responses (probable bot automation). Manipulation checks (MC) [31] and a control question in the 5-Trial adjusting delay discounting task assessed a basic level of attention. The first MC, "As a child, were you raised by an old woman in a shoe?" was placed in the demographic questionnaire, the second MC, "Being eaten by a grizzly bear…" was placed in the QOLS pre-COVID condition, and the third MC, "Your nose growing 6 inches overnight…" was placed in the QOLS during-COVID condition. The control choice, "Which is greater, $4.74 OR $7.47?" was placed in the 5-Trial adjusting delay discounting task. Two of three correct manipulation check responses and the correct control choice were required for data inclusion. Suspiciously fast completion times indicated either gross inattention or likely bot automation. In-house testing (*n* = 6) indicated that the Recovery and Active User surveys required >20 minutes each, with some individual variation producing longer completion times. This informed our minimum time completion threshold to exclude times < 900 seconds (15 minutes) as probable bots or highly inattentive participants (exclusions: Recovery = 241, Active User = 333).

*Resilience* was assessed using the 10-item Connor-Davidson Resilience Scale (CD-RISC-10), a short version adapted from the original 25-item scale (good reliability; α = 0.853). Total CD-RISC-10 scores range from 0 to 40, with higher scores indicating greater resilience [16].

*Self-reported Impulsivity* was assessed using the English short-version Urgency (α = 0.800), Lack of Premeditation (α = 0.815), Lack of Perseverance (α = 0.753), Sensation Seeking (α = 0.667), Positive Urgency (α = 0.845), Impulsive Behavior Scale (SUPPS-P). The SUPPS-P [20] has four items per subscale and is scored on a 4-point Likert-type scale from 1 = Agree Strongly to 4 = Disagree Strongly (including some reverse-coded items), with higher scores indicating greater impulsivity.

*Discounting behavior* was assessed using the 5-Trial Adjusting Delay Discounting Task implemented in Qualtrics. The task quantifies temporal discounting of money (i.e., "Which would you prefer, $50 now or $100 in 3 weeks?") in less than 1 minute and generates good reliability with longer-form discounting tasks [32]. Unlike the more common adjusting amount tasks that adjust the immediate amount (e.g., [21]), the 5-trial adjusting delay presents pre-determined delays indexing discounting rates, with amounts held constant, i.e., adjusting delay. Discounting behavior was quantified as *k* values, which were normalized by log transform for parametric analyses.

*Quality of life* was assessed using a 9-item Quality-of-Life Scale (QOLS) adapted from the original 16-item version [33]. The QOLS is determined by evaluating participant responses in life domains ranging from physical and material well-being to relations with others. Items 3–8 (e.g., “learning,” “understanding yourself,” “work,” “expressing yourself,” “reading,” and “participating in recreation”), and 13 (“socializing”) were removed from the QOLS to eliminate aspects of interpersonal relationships to reduce participant burden. The remaining nine items on the QOLS were scored on a 7-point Likert scale from 7 = Delighted to 1 = Terrible (α = 0.898). Higher QOLS scores indicate a greater perceived quality of life [33]. The QOLS was administered once for the pre-COVID condition and once for the during-COVID condition.

*Alcohol and illicit substance use* were evaluated using an adapted 45-day timeline follow back from the National Institute of Drug Abuse Timeline Followback (NIDA TLFB). The TLFB was administered once for the pre-COVID condition and once for the during-COVID condition. Participants’ weekly alcohol use amount was assessed by dividing the total number of standard drinks consumed in each 45-day time period by number of weeks (i.e., 6.43). If participants endorsed using more than three illicit substances, they were prompted to select the three most used for each time period. Participants’ weekly substance use amount was quantified by converting all units (except for marijuana and inhalants) to milligrams, then averaging the substance used at each time period. Marijuana and inhalants were quantified as "1 use event = consumed/inhaled substance and got high/intoxicated" and averaged at each time period. Frequency of alcohol and substance use was measured by averaging the number of days used at each time period.

*Life events* were assessed using items from the Pathways Baseline Assessment [15]. Items representing life events were administered once for the pre-COVID condition and once for the during-COVID condition. Life event items required participants to select from a list of 13 possible experiences (i.e., death of a loved one, divorce/separation, trouble with the law, personal injury or illness, injury/illness of a loved one, serious problem with work/school, serious financial difficulty, loss of employment, increased responsibility, change/start work or school, change in living conditions, a victim of a crime/violence/accident, and not applicable [N/A]) they may have had during each time period, and then rate how that experience impacted them on a 5-point Likert-type scale from 0 = Not at all to 4 = Extremely.

*Recovery activity and involvement* were assessed using items from the Pathways Baseline Assessment for the Recovery group [15]. Items pertaining to recovery activities and involvement were administered once for the pre-COVID condition and once for the during-COVID condition. Participants were queried about drug and alcohol use, the substance(s) used, the day of last use, the number of use events, and the amount consumed. In addition, participants selected support group involvement (Narcotics Anonymous, Alcoholics Anonymous, Cocaine Anonymous, Substance Treatment and Recovery, Secular Organization for Sobriety, Rational Recovery, Moderation Management, Other, and None) for both time periods, then specified their level of involvement (including having or being a sponsor/mentor for each time period).

### 2.3. Analysis

All data were analyzed and checked for normality in SPSS (v27; IBM). Only Delay Discounting *k*-values required transformation (log[10]) for normality. Reliability was reported for all self-report assessments (*n* = 79) as Cronbach’s alpha. Paired *t*-tests examined differences within groups in alcohol and substance use, the number of life events, and recovery activities (in the early recovery participants). Pandemic-induced change in the impact of life events was tested in a Time × Life Event repeated measures ANOVA (Greenhouse-Geisser-corrected for non-sphericity), with significant interactions followed by paired *t*-tests. Means and standard deviations are reported as (*M* ± *SD*) in text and tables. Pearson correlations were conducted for exploratory comparisons. All *t*-tests were two-tailed. Alpha was set to 0.05, with protection against Type I error from multiple comparisons where appropriate (Benjamini-Hochberg false discovery rate *q* < .05). Demographics (age, biological sex, race, ethnicity, education, income, and nicotine statistics) are detailed in Table 1. Ancillary data are provided in *Supporting Information*.

**Table 1:** Demographics.

	Active User (*n* = 45)		Early Recovery (*n* = 34)	
	*M* ± *SD*	*n*(%)	*M* ± *SD*	*n*(%)
Age	37 ± 11.5	-	36 ± 9.8	-
Male	-	32(71)	-	20(59)
Female	-	13(29)	-	14(41)
White/Caucasian	-	32(71)	-	28(82)
Black/African American	-	5(11)	-	4(12)
Native American/Eskimo	-	4(9)	-	1(3)
Asian	-	4(9)	-	-
Bi-racial^a^	-	-	-	1(3)
Hispanic/Latino	-	2(4)	-	7(21)
Bachelor’s Degree	-	24(53)	-	21(62)
Childhood Household Income	$49,000^**b**^	-	$49,000^**b**^	-
Monthly Disposable Income	$500^**b**^	-	$1,000^**b**^	-
Current Student	-	9(20)	-	10(29)
Daily Smoker (cigarettes)	-	27(60)	-	22(65)

^a^Self-Identified as White/Caucasian and Native American/Eskimo

^b^Median

## 3. Results

*Demographics*. The majority of MTurk participants in this study were white (76%) and male (66%), ages 37 ± 8 (Table 1). Eighteen percent of MTurk participants identified California as their primary residence, followed by Florida at 10% (Table 2).

**Table 2:** Geographic distribution by group.

State	Active User (*n* = 45)	Early Recovery (*n* = 34)
	*n*(%)	*n*(%)
California	10(22)	4(12)
Connecticut	2(4)	-
Florida	3(7)	4(12)
Georgia	3(7)	-
Hawaii	1(2)	-
Illinois	1(2)	3(9)
Kansas	1(2)	-
Kentucky	1(2)	-
Maine	-	1(3)
Maryland	1(2)	-
Michigan	1(2)	-
Missouri	1(2)	-
Nevada	1(2)	3(9)
New Jersey	-	3(9)
New York	5(11)	1(3)
North Carolina	3(7)	2(6)
Ohio	3(7)	3(9)
Oklahoma	-	1(3)
Pennsylvania	-	2(6)
South Carolina	1(2)	1(3)
Tennessee	2(4)	-
Texas	3(7)	3(9)
Utah	-	1(3)
Virginia	1(2)	1(3)
Washington	1(2)	1(3)

Self-reported location (state) was verified with IP2 Location Services (see 2.1. Participants).

### 3.1. Active users

#### Alcohol and illicit substance use

Weekly drinking amount significantly decreased during- versus pre-COVID (15.38 ±21.74 and 22.05 ±23.25, respectively), *t*(44) = 2.91, *p* = .006, *q* < .05. Frequency or the number of days spent consuming alcohol (27.24 ±18.87, and 32.78 ±16.41) and most-used illicit substance, predominately cannabis (18.46 ±17.13, and 26.54 ±16.60) also decreased during- versus pre-COVID, *t*(44) = −2.62, *p* = .012, and *t*(12) = −2.26, *p* = .043, *q* < .05, respectively (Table 3). No other significant changes in illicit substance use were observed. Illicit substance use is characterized in Table 4.

**Table 3:** Pandemic-related change in use of alcohol and illicit substances in active users.

	Active User (*n* = 45)	
	*M* ± *SD*	(*n*)
Weekly alcohol consumption	−6.67 ± 15.37*	45
Days spent drinking alcohol	−5.53 ± 14.19*	45
Days spent using 1st illicit substance^a^	−8.08 ± 12.86*	27

^a^Substanaces were rank-ordered according to amount of use per week (88% cannabis)

*q < .05

Means and standard deviations are reported as difference scores (during-COVID−pre-COVID)

**Table 4:** Illicit substance use by category pre-COVID and during-COVID among active users.

	Active User (n = 45)	
	Pre-COVID	During-COVID
	*n*(%)	*n*(%)
Cannabis/Marijuana	20(44)	15(33)
Cocaine/Crack	3(6)	1(2)
MDMA/Ecstasy	-	-
Amphetamine/Methamphetamine	1(2)	1(2)
Opioid analgesics (including methadone)	1(2)	1(2)
Heroin	3(6)	1(2)
Hallucinogens	-	-
Sedatives/Hypnotics (excluding Benzodiazepines)	1(2)	-
Benzodiazepines	-	-
Inhalants	1(2)	-

The majority of participants (64%) endorsed use of illicit substances pre-COVID, compared to 41% during-COVID. Cannabis/Marijuana was the highest selected substance of choice at both time periods.

#### Resilience

The mean CD-RISC-10 score was 24.16 ± 6.90; there was no correlation between resilience and the change in the amount or frequency of alcohol and substances used during- versus pre-COVID, *q*>.05, (Table 5).

**Table 5:** Resilience and pandemic-related change in alcohol/drug use in active users.

	Active User (*n* = 45)
	*r*
Weekly alcohol consumption	.25
Days spent consuming alcohol	.22
Number of substances used	−.07
Days spent using 1^st^ illicit substance^a^	−.24

^a^Only 1^st^ illicit substance choice was listed due to small sample size

Means and standard deviations are reported as difference scores (during-COVID−pre-COVID)

#### Life events

Although the Time × Life Event interaction was significant, *F*(6.3,276.7) = 3.11, *p* = .005, we did not detect differences in individual life events that survived FDR correction (Table 6).

**Table 6:** Self-reported life events pre-COVID and during-COVID among active users.

	Active User (*n* = 45)	Pre-COVID	During-COVID
	*M* ± *SD*	*n(%)*	*n(%)*
**Number of life events**	−0.11 ± 1.48		
Death of a loved one	−0.38 ± 1.17	6(13)	^c^
Divorce/Separation	0.04 ± 0.29	^b^	1(2)
Trouble with the law	0.24 ± 0.80	1(2)	3(7)
Personal injury or illness	−0.35 ± 1.09	13(29)	5(11)
Injury/illness of a loved one	−0.20 ± 1.05	4(9)	2(4)
Problems with work/school	−0.04 ± 1.10	8(18)	6(13)
Financial difficulties	0.51 ± 1.53	11(24)	18(40)
Loss of employment	−0.31 ± 0.95	7(15)	2(4)
Increased responsibility	−0.18 ± 1.43	14(32)	9(20)
Changing/starting work/school	0.13 ± 0.55	1(2)	4(9)
Changes in living conditions	0.02 ± 1.07	6(13)	5(11)
Victim of crime, violence, or accident	^a^	^a^	^a^

^a^No self-reported victim of crime, violence, or accident

^b^No self-reported divorce/separation

^c^No self-reported death of a loved one

Means and standard deviations are reported as difference scores (during-COVID−pre-COVID)

#### Quality of life

No significant differences were observed in QOL during- versus pre-COVID.

#### Impulsivity and discounting behavior

There were no significant correlations between pandemic-related changes in alcohol/substance use (amount, frequency, and number) and impulsivity (DD and SUPPS-P), *q*>.05, (Table 7). Lack of premeditation (1.76 ±0.52, *r*(45) = 0.41, *p* = .005, *q* < .05) correlated with drinks/week pre-COVID. Substance use (amount and frequency) was uncorrelated, *q*>.05.

**Table 7:** Impulsivity and pandemic-related change in alcohol and substance use in active users.

	Active User (*n* = 45)	Δ Alcohol Amount	Δ Alcohol Frequency	Δ Number of Substances	Δ Substance Use Frequency
	*M* ± *SD*	*r*	*r*	*r*	*r*
Delay Discounting *k-*value^a^	−1.26 ± 1.38	−.04	−.11	−.23	−.20
*SUPPS-P Subscales*					
Negative Urgency	2.53 ± 0.78	−.09	−.17	−.17	.20
Lack of Perseverance	1.72 ± 0.54	−.12	−.23	−.05	.10
Lack of Premeditation	1.76 ± 0.52	−.16	−.24	.04	.04
Sensation Seeking	2.56 ± 0.64	-.04	−.13	−.02	.20
Positive Urgency	2.22 ± 0.84	−.03	−.05	−.12	.15

^a^loag(10) transformed

### 3.2 Early recovery

#### Recovery activity and involvement

Recovery group attendance decreased during- versus pre-COVID (0.79 ±0.88 and 1.15 ±0.89, respectively; 31% decline) as a trend *p* = .032, but did not survive FDR correction (Table 8). Use events did not differ during- versus pre-COVID *q>*.05.

**Table 8:** Pandemic-related change in use events and recovery involvement.

	Early Recovery (*n* = 34)
	*M* ±*SD*
Use Events	0.21 ± 0.64
Recovery Group Involvement	−0.35 ± 0.92
Sponsor/Mentor in Recovery Group	−0.11 ± 0.51

Means and standard deviations are reported as difference scores (during-COVID−pre-COVID)

#### Resilience

Greater resilience (CD-RISC-10 mean score; 25.56 ± 5.71) correlated with the change in use, *r*(34) = −.50, *p* = .006, *q* < .05, (Table 9), i.e., people with greater resilience showed smaller pandemic-related alterations in use patterns.

**Table 9:** Resilience and pandemic-related change in use and recovery activities.

	Early Recovery (*n* = 34)	
	*M* ± *SD*	*r*
Use events	0.21 ± 0.64	−.50*
Recovery group attendance	−0.26 ± 0.86	−.29
Sponsor/mentor in recovery group	−0.11 ± 0.51	.17

*q < .05

Means and standard deviations are reported as difference scores (during-COVID−pre-COVID)

#### Life events

The Time × Life Event interaction was significant, *F*(4.8,154.1) = 4.62, *p* < .001, (although responsibility indicated significance, the small *n* compromises meaningful interpretation); Table 10.

**Table 10:** Self-reported life events.

	Early Recovery (*n* = 34)	Pre-COVID	During-COVID
	*M* ± *SD*	*n(%)*	*n(%)*
**Number of life events**	−0.21 ± 1.23		
Death of a loved one	0.06 ± 0.34	1(2)	2(4)
Divorce/Separation	0.03 ± 0.17	1(2)	1(2)
Trouble with the law	0.09 ± 0.38	1(2)	2(4)
Personal injury or illness	−0.29 ± 1.22	11(24)	3(7)
Injury/illness of a loved one	0.03 ± 1.14	3(7)	4(9)
Problems with work/school	0.06 ± 0.85	4(9)	15(33)
Financial difficulties	0.68 ± 1.77	10(22)	1(2)
Loss of employment	−0.32 ± 0.88	5(11)	9(20)
Increased responsibility	−0.91 ± 1.56*	14(32)	2(4)
Changing/starting work/school	−0.15 ± 0.74	20(44)	8(18)
Changes in living conditions	0.18 ± 1.19	5(11)	5(11)
Victim of crime, violence, or accident	0.03 ± 0.17	^a^	1(2)

^a^No self-reported victim of crime, violence, or accident

Means and standard deviations are reported as difference scores (during-COVID−pre-COVID)

#### Quality of life

There was a significant overall reduction in QOL during- versus pre-COVID (44.88 ±8.27 and 47.47 ±6.65, respectively), *t*(33) = −2.32, *p* = .027.

#### Impulsivity and discounting behavior

The change in number of use events during- versus pre-COVID was uncorrelated with DD and SUPPS scores, *q*>.05, (Table 11).

**Table 11:** Impulsivity and pandemic-related change in use events among the recovery group.

	Early Recovery (*n* = 34)	Δ Use Events
	*M* ± *SD*	*r*
Delay Discounting *k-*value^a^	−0.76 ± 1.21	−0.26
*SUPPS-P Subscales*		
Negative Urgency	2.53 ± 0.64	−0.14
Lack of Perseverance	1.74 ± 0.43	0.23
Lack of Premeditation	1.78 ± 0.41	0.003
Sensation Seeking	2.63 ± 0.71	−0.21
Positive Urgency	2.32 ± 0.70	−0.14

^a^*k*-values reported as log(10) transformed

### 3.3. Excluded data

There were 49 participants in the Active User group and 64 participants in the Recovery group excluded from the main analyses. While we believed these to be actual human participants, and thus informative data, their unknown locations required separate analyses for US-relevant interpretations (see section 2.1 Participants for more information). Results for these participants are reported in supporting information tables (S1–S10 Tables).

## 4. Discussion

We quantified outcomes before and early in the pandemic to better understand pandemic-related changes in alcohol/drug use, recovery outcomes, and quality of life in addition to interactions with personality and behavioral traits. Importantly, we assessed time periods uncontaminated by the sociopolitical unrest beginning in the USA in late May 2020 by using our “during” pandemic assessment period ending May 15^th^, 2020. The first hypothesis, that there would be increased use of alcohol and illicit substances, including marijuana among the active user group during-COVID, was not supported. The amount of alcohol consumption decreased as well as the number of days spent using alcohol and illicit substances. However, the number of substances used during-COVID was not statistically different from pre-COVID. The second hypothesis was similarly unsupported, as use events during-COVID did not significantly increase among the Recovery group. The third hypothesis was partially supported, as engagement in recovery activities substantially declined during the pandemic (falling short of FDR correction). The fourth hypothesis was also unsupported, as higher DD and total SUPPS-P scores were not correlated with increased use events among the Active User and Recovery groups. Finally, the fifth hypothesis was partially supported—in the Recovery group, resilience correlated with better outcomes, with more-resilient participants showing less pandemic-related change in use. The most notable effects were large declines in recovery activities in the Recovery group and reduced use in the Active User group; both of which were likely possible outcomes of stay-at-home measures.

The overall effect of the pandemic on drinking is still unclear, despite hundreds of thousands of participants reporting drinking data. Large meta-analyses suggest inconsistent findings, with one reporting no mean effect (aggregate *N* = 492,235), but altered drinking in about half of participants, i.e., 23% decreased, 23% increased [6]. Another study (*N* = 259,188) showed decreased (consumption in Australia) or increased (frequency of alcohol use in the US and proportion of problem drinkers in the UK) [7]. Both studies suggested country and income-specific differences in the pandemic’s effect on drinking. In Europe, more individuals reported decreases in alcohol use and frequency than increases [34]. Mechanisms driving increased use of alcohol and/or drugs include economic worries and isolation [35] and mental health factors [36]. The duration of isolation appeared to exert powerful effects on alcohol use severity, with AUDIT scores increasing after just two months into lockdowns, and nearly doubling by September 2020 [37]. Similar to the current study (reporting decreased alcohol and substance use) strong effects took months to emerge. The weight of evidence does not support clear predictions about pandemic effects on alcohol and drug use, as they vary widely according to region, individual prior use patterns, economic status, mental health, and isolation policy.

Self-reported QOL declined during-COVID among the Recovery group. No significant changes in QOL were observed among the Active User group, which could be the result of a floor effect, since Active User’s pre-COVID QOL was lower than the Recovery group, *t*(77) = −2.44, *p* = .017. The QOL reduction among the Recovery group could be related to interrupted access to support services during the pandemic, which decreased (pre- versus during-pandemic support group involvement. Prior work has also found similar results regarding restricted access to treatment and general healthcare provider services [38]. Relatedly, the rapid transition of recovery activities to an online format (as opposed to the usual in-person) may also explain this result [38]. Despite visits doubling during the pandemic, online recovery support services may not have the same impact on recovery outcomes compared to in person formats [39–41]. The QOL decrement was not explained by an altered number of life events, as we did not detect changes in either group. While the availability of telehealth options may have provided much-needed support in the early days of the pandemic, this protection may have rapidly waned, and perhaps inverted, comporting with the observed initial decrease, then increase in psychiatric and substance use-related emergency room visits [42, 43].

Individual personality differences can also account for study findings. Resilience is the resistance to the maladaptive effects of stress and trauma, and protection against precipitated psychiatric dysfunction in the presence of adversity [44, 45]. It is likely to be particularly relevant in the context of pandemic-related adversity effects on alcohol/drug use and recovery maintenance. However, given this assumption, it is surprising resiliency did not track with use among the Active User group as it did among the Recovery group (negative correlation). As such, only self-reported impulsivity (lack of premeditation) correlated with weekly alcohol consumption among the Active User group, no other correlations of self-reported impulsivity were found in either group. Behavioral impulsivity (delay discounting) was uncorrelated with pandemic-induced change in alcohol/drug use in both groups. Resilience and impulsivity may have interacted with certain endophenotypes or other affective states that were not assessed in this study.

MTurk is a convenient method for rapidly collecting large data sets. However, it is not without its hazards, as clearly illustrated by this study. Only 5% of our collected data passed all fidelity checks. Despite the purported Amazon enforcement of our specified inclusion criterion of “US residents only”, suspicion was aroused during the initial data collection. Responses in fillable fields often appeared to reflect non-native English grammar/syntax. This led to our final filtration analysis using IP physical locations (IP2location.com), which confirmed that 19% of participants who passed our stringent fidelity checks were likely performing study procedures from outside the US, leaving us with our final sample of 79 participants. The prevalence of bots and bad actors adds uncontrolled and unwanted variability to any data collection enterprise. While likely common to any internet-based data collection tool, the widespread involvement of untruthful enrollees was deeply concerning and should be carefully considered by any researchers attempting this method of data collection [46–48]. We believed our MTurk data set could be trusted only after stringent filtering and analysis to confirm attention, comprehension, and IP physical location. Overlooking the untruthful location reporting, we believed that the data passing all fidelity checks (except location) still provided a useful comparison for non-US pandemic effects. These ancillary data produced findings that resembled our trusted US-based data set, particularly regarding use (S2 Table) and recovery (S7 Table).

A major strength of this work was isolating the time periods under study to those isolated to viral pandemic effects and unaffected by confounding sociopolitical-related upheaval. While the pandemic elicited global fear, the civil unrest arising months into the pandemic varied substantially across the globe. Late May of 2020 in the United States was a particularly challenging time, with the combined effects of COVID-19 and the civil unrest relating to policing and racial tensions [49]. We recommend that research focused specifically on COVID-19-mediated effects carefully consider the confounding effects of the social unrest overlapping this time period.

Some limitations warrant consideration. The final sample analyzed was modest. This resulted from quality screening that eliminated 95% of the total number initially recruited. We believe rigorous screening of data collected online is critical to drawing meaningful conclusions and suspect that much of the published work using online data sets falls short on fidelity checking. Our sample was an imperfect cross-section of the US population, as it was skewed toward male and white participants—possibly an artifact of online study interest. The (necessarily) online delivery of the study measures includes several limitations intrinsic to web-based surveys, including suboptimal precision from careless responding [50, 51] and biased population sampling [52, 53]. These include requirements of a computer and stable internet, which may pose obstacles for some lower socioeconomic status active and recovering ASUD individuals [54–56]. Finally, while we attempted to minimize the participant burden for the current study, it is possible that the completion of two TLFBs and recovery involvement assessments may have generated some fatigue and contributed to careless responding. Unfortunately, the pandemic’s increase in the number of people physically isolated, online, and potentially unemployed may have further incentivized fraudulent study participation. While we believe the time period under study was a strength, it is also a limitation when attempting to extrapolate to the”pandemic effects” writ large—as noted previously regarding variation by time period sampled [37]. We advise caution in generalizing these current findings to the US population in light of the current limitations.

## 5. Conclusions

The COVID-19 pandemic produced the greatest societal upheaval in a generation, with its effects on alcohol/drug use and recovery still being understood. Our findings suggest that pandemic policies, including stay-at-home orders, may have differentially impacted those in early recovery. While we detected reduced use in current users, this was apparently temporary as other work has shown overall increased use [36, 43, 57] coinciding with accelerated deaths due to alcohol and overdose [42, 58, 59]. From a public health standpoint, the ramifications of stay-at-home orders on mental health and addiction must be carefully weighed in the cost/benefit tradeoff for preventing communicable disease transmission.

## Supporting information

S1 Table Ancillary data, demographics. (DOCX)

S2 Table Ancillary data, pandemic-related change in use of alcohol in active users. (DOCX)

S3 Table Ancillary data, Illicit substance use by category among active users. (DOCX)

S4 Table Ancillary data, resilience and pandemic-related change in alcohol/drug use in active users. (DOCX)

S5 Table Ancillary data, pandemic-related change in active User life events and impact ratings. (DOCX)

S6 Table Ancillary data, impulsivity and pandemic-related change in alcohol use in active users. (DOCX)

S7 Table Ancillary data, pandemic-related change in use events and recovery involvement. (DOCX)

S8 Table Ancillary data, resilience and pandemic-related change in use and recovery activities. (DOCX)

S9 Table Ancillary data, pandemic-related change in recovery group life events and impact ratings. (DOCX)

S10 Table Ancillary data, impulsivity and pandemic-related change in Recovery group use events. (DOCX)

S1 File (DOCX)
