# Supplementary material for: Changes in substance use, recovery, and quality of life during the initial phase of the COVID-19 pandemic
Source: PLoS One. 2024 May 22;19(5):e0300848. doi: 10.1371/journal.pone.0300848 (PMC11111065; doi:10.1371/journal.pone.0300848)
Supplement: S1 Table — (DOCX) [file pone.0300848.s001.docx]

| S1 Table.  Ancillary Data^a^, Demographics | | | | | |  | |  | |  |  |  |  |  |
| --- | --- | --- | --- | --- | --- | --- | --- | --- | --- | --- | --- | --- | --- | --- |
|  | **Active User**  **(*n* = 49)** | | | **Early Recovery**  **(*n* = 64)** | | | | |  |  |  |  |  |  |
|  | *M* ± *SD* |  | *n*(%) | *M* ± *SD* |  | | *n*(%) | |  |  |  |  |  |  |
| Age | 36 ± 11.8 |  | - | 36 ± 9.8 |  | | - | |  |  |  |  |  |  |
| Male | - |  | 38(78) | - |  | | 20(59) | |  |  |  |  |  |  |
| Caucasian | - |  | 39(80) | - |  | | 28(82) | |  |  |  |  |  |  |
| Hispanic/Latino | - |  | 13(27) | - |  | | 13(20) | |  |  |  |  |  |  |
| Bachelor’s Degree | - |  | 20(41) | - |  | | 21(62) | |  |  |  |  |  |  |
| Childhood Household Income | $49,000* |  | - | $49,000* |  | | - | |  |  |  |  |  |  |
| Monthly Disposable Income | $2,000* |  | - | $2,000* |  | | - | |  |  |  |  |  |  |
| Current Student | - |  | 17(35) | - |  | | 23(36) | |  |  |  |  |  |  |
| Daily Smoker (cigarettes) | - |  | 38(78) | - |  | | 59(92) | |  |  |  |  |  |  |
| *Median value  ^a^Participants excluded from main analyses due to inability to verify US location | | | | | | | | | | | | | | |
