# Supplementary material for: Changes in substance use, recovery, and quality of life during the initial phase of the COVID-19 pandemic
Source: PLoS One. 2024 May 22;19(5):e0300848. doi: 10.1371/journal.pone.0300848 (PMC11111065; doi:10.1371/journal.pone.0300848)
Supplement: S2 Table — (DOCX) [file pone.0300848.s002.docx]

| **S2 Table.**  **Ancillary Data^a^, Pandemic-related change in use of alcohol in active users** | | | |
| --- | --- | --- | --- |
|  | **Active User (*n* = 49)** | |  |
|  | *M* ± *SD* | (*n*) |  |
| Weekly alcohol consumption | −0.99 ± 6.33 | 49 |  |
| Days spent drinking alcohol | −2.22 ± 10.51 | 49 |  |
| ^a^Participants excluded from main analyses due to inability to verify US location  Means and standard deviations are reported as difference scores (during-COVID−pre-COVID)  Note that illicit substance use was uncommon, with only 9 participants reporting any use pre- or during-COVID (8 out of 9 were cannabis). | | | |
