# Supplementary material for: Changes in substance use, recovery, and quality of life during the initial phase of the COVID-19 pandemic
Source: PLoS One. 2024 May 22;19(5):e0300848. doi: 10.1371/journal.pone.0300848 (PMC11111065; doi:10.1371/journal.pone.0300848)
Supplement: S3 Table — (DOCX) [file pone.0300848.s003.docx]

| **S3 Table.**  **Ancillary Data^a^, Illicit substance use by category among active users** | | | | |
| --- | --- | --- | --- | --- |
|  | **Active User (*n* = 49)** | | |  |
|  | **Pre-COVID** | | **During-COVID** |  |
|  | *n*(%) | | *n*(%) |  |
| Cannabis/Marijuana | 6(12) | 6(12) | |  |
| Cocaine/Crack | 3(6) | 2(4) | |  |
| MDMA/Ecstasy | - | 1(2) | |  |
| Amphetamine/Methamphetamine | 1(2) | - | |  |
| Opioid analgesics (including methadone) | - | - | |  |
| Heroin | 1(2) | 1(2) | |  |
| Hallucinogens | 1(2) | - | |  |
| Sedatives/Hypnotics (excluding Benzodiazepines) | - | - | |  |
| Benzodiazepines | - | - | |  |
| Inhalants | - | 1(2) | |  |
| ^a^Participants excluded from main analyses due to inability to verify US location | | | | |
