# Supplementary material for: Changes in substance use, recovery, and quality of life during the initial phase of the COVID-19 pandemic
Source: PLoS One. 2024 May 22;19(5):e0300848. doi: 10.1371/journal.pone.0300848 (PMC11111065; doi:10.1371/journal.pone.0300848)
Supplement: S4 Table — (DOCX) [file pone.0300848.s004.docx]

| **S4 Table.**  **Ancillary Data^a^, Resilience and pandemic-related change in alcohol/drug use in active users** | | | |
| --- | --- | --- | --- |
|  | **Active User (*n* = 49)** | |  |
|  | *r* |  |  |
| Weekly alcohol consumption | .20 |  |  |
| Days spent consuming alcohol | .09 |  |  |
| Number of substances used | .20 |  |  |
| Days spent using 1^st^ illicit substance | .25 |  |  |
| ^a^Participants excluded from main analyses due to inability to verify US location | | | |
