# Supplementary material for: Changes in substance use, recovery, and quality of life during the initial phase of the COVID-19 pandemic
Source: PLoS One. 2024 May 22;19(5):e0300848. doi: 10.1371/journal.pone.0300848 (PMC11111065; doi:10.1371/journal.pone.0300848)
Supplement: S5 Table — (DOCX) [file pone.0300848.s005.docx]

| **S5 Table.**  **Ancillary Data^a^, Pandemic-related change in Active User life events and impact ratings** | | |
| --- | --- | --- |
|  | **Active User**  **(*n* = 49)** |  |
|  | *M* ± *SD* |  |
| Number of life events | −0.06 ± 0.66 |  |
| Death of a loved one | −0.41 ± 1.02 |  |
| Divorce/Separation | 0.02 ± 0.72 |  |
| Trouble with the law | 0.02 ± 0.52 |  |
| Personal injury or illness | −0.02 ± 1.22 |  |
| Injury/illness of a loved one | 0.14 ± 1.26 |  |
| Problems with work/school | 0.22 ± 0.87 |  |
| Financial difficulties | 0.00 ± 1.44 |  |
| Loss of employment | −0.10 ± 0.65 |  |
| Increased responsibility | 0.10 ± 1.16 |  |
| Changing/starting work/school | −0.06 ± 0.47 |  |
| Changes in living conditions | 0.06 ± 1.01 |  |
| Victim of crime, violence, or accident | −0.06 ± 0.43 |  |
| ^a^Participants excluded from main analyses due to inability to verify US location  Means and standard deviations are reported as difference scores (during-COVID−pre-COVID) | | |
