# Supplementary material for: Changes in substance use, recovery, and quality of life during the initial phase of the COVID-19 pandemic
Source: PLoS One. 2024 May 22;19(5):e0300848. doi: 10.1371/journal.pone.0300848 (PMC11111065; doi:10.1371/journal.pone.0300848)
Supplement: S6 Table — (DOCX) [file pone.0300848.s006.docx]

| **S6 Table.**  **Ancillary Data^a^, Impulsivity and pandemic-related change in alcohol use in active users** | | | | |  |
| --- | --- | --- | --- | --- | --- |
|  | **Active User**  **(*n* = 49)** | **Δ Alcohol Amount** | **Δ Alcohol Frequency** |  |  |
|  | *M* ± *SD* | *r* | *r* |  |  |
| Delay Discounting  *k-*value^b^ | −0.91 ± 1.15 | 0.061 | −0.044 |  |  |
|  |  |  |  |  |  |
| *SUPPS-P Subscales* |  |  |  |  |  |
| Negative Urgency | 2.61 ± 0.62 | −0.174 | −0.146 |  |  |
| Lack of Perseverance | 1.70 ± 0.47 | −0.066 | −0.107 |  |  |
| Lack of Premeditation | 1.81 ± 0.49 | −0.212 | −0.017 |  |  |
| Sensation Seeking | 2.80 ± 0.78 | −0.181 | −0.127 |  |  |
| Positive Urgency | 2.34 ± 0.78 | −0.149 | −0.228 |  |  |
| ^a^Participants excluded from main analyses due to inability to verify US location  ^b^log(10) transformed | | | | | |
