# Supplementary material for: Changes in substance use, recovery, and quality of life during the initial phase of the COVID-19 pandemic
Source: PLoS One. 2024 May 22;19(5):e0300848. doi: 10.1371/journal.pone.0300848 (PMC11111065; doi:10.1371/journal.pone.0300848)
Supplement: S7 Table — (DOCX) [file pone.0300848.s007.docx]

| **S7 Table.**  **Ancillary Data^a^, Pandemic-related change in use events and recovery involvement** | | |
| --- | --- | --- |
|  | **Early Recovery**  **(*n* = 64)** |  |
|  | *M* ± *SD* |  |
| Use Events | −0.17 ± 0.46^b^ |  |
| Recovery Group Involvement | −0.27 ± 0.84^c^ |  |
| Sponsor/Mentor in Recovery Group | 0.02 ± 0.60 |  |
| ^a^Participants excluded from main analyses due to inability to verify US location  ^b^*t*(63)= −3.01, *p*=.004  ^c^*t*(63)= −2.53, *p*=.014  Means and standard deviations are reported as difference scores (during-COVID−pre-COVID) | | |
