# Supplementary material for: Changes in substance use, recovery, and quality of life during the initial phase of the COVID-19 pandemic
Source: PLoS One. 2024 May 22;19(5):e0300848. doi: 10.1371/journal.pone.0300848 (PMC11111065; doi:10.1371/journal.pone.0300848)
Supplement: S8 Table — (DOCX) [file pone.0300848.s008.docx]

| \| **S8 Table.**  **Ancillary Data^a^, Resilience and pandemic-related change in use and recovery activities** \| \| --- \| | | | |
| --- | --- | --- | --- | --- |
|  | **Early Recovery**  **(*n* = 64)** | |  |
|  | *M* ± *SD* | *r* |  |
| Use events | −0.17 ± 0.05 | −.08 |  |
| Recovery group attendance | −0.27 ± 0.84 | .09 |  |
| Sponsor/mentor in recovery group | 0.02 ± 0.60 | .21 |  |
| ^a^Participants excluded from main analyses due to inability to verify US location  Means and standard deviations are reported as difference scores (during-COVID−pre-COVID) | | | |
