# Supplementary material for: Changes in substance use, recovery, and quality of life during the initial phase of the COVID-19 pandemic
Source: PLoS One. 2024 May 22;19(5):e0300848. doi: 10.1371/journal.pone.0300848 (PMC11111065; doi:10.1371/journal.pone.0300848)
Supplement: S9 Table — (DOCX) [file pone.0300848.s009.docx]

| **S9 Table.**  **Ancillary Data^a^, Pandemic-related change in Recovery group life events and impact ratings** | | |
| --- | --- | --- |
|  | **Early Recovery**  **(*n* = 64)** |  |
|  | *M* ± *SD* |  |
| Number of life events | −0.34 ± 1.21 |  |
| Death of a loved one | −0.41 ± 1.35 |  |
| Divorce/Separation | −0.02 ± 0.45 |  |
| Trouble with the law | −0.09 ± 1.00 |  |
| Personal injury or illness | 0.00 ± 1.23 |  |
| Injury/illness of a loved one | −0.11 ± 1.20 |  |
| Problems with work/school | 0.08 ± 1.12 |  |
| Financial difficulties | −0.33 ± 1.08 |  |
| Loss of employment | −0.05 ± 1.13 |  |
| Increased responsibility | −0.14 ± 1.39 |  |
| Changing/starting work/school | −0.20 ± 1.05 |  |
| Changes in living conditions | 0.02 ± 1.24 |  |
| Victim of crime, violence, or accident | 0.08 ± 0.45 |  |
| ^a^Participants excluded from main analyses due to inability to verify US location  Means and standard deviations reported as difference scores (during-COVID−pre-COVID) | | |
