# Supplementary material for: Changes in substance use, recovery, and quality of life during the initial phase of the COVID-19 pandemic
Source: PLoS One. 2024 May 22;19(5):e0300848. doi: 10.1371/journal.pone.0300848 (PMC11111065; doi:10.1371/journal.pone.0300848)
Supplement: S10 Table — (DOCX) [file pone.0300848.s010.docx]

| **S10 Table.**  **Ancillary Data^a^, Impulsivity and pandemic-related change in Recovery group use events** | | | |
| --- | --- | --- | --- |
|  | **Early Recovery (*n*=64)** | **Δ Use Events** |  |
|  | *M* ± *SD* | *r* |  |
| Delay Discounting  *k-*value^b^ | −0.76 ± 1.40 | −0.10 |  |
|  |  |  |  |
| *SUPPS-P Subscales* |  |  |  |
| Negative Urgency | 2.57 ± 0.54 | 0.04 |  |
| Lack of Perseverance | 1.83 ± 0.50 | −0.31* |  |
| Lack of Premeditation | 1.74 ± 0.55 | −0.12 |  |
| Sensation Seeking | 2.76 ± 0.57 | 0.04 |  |
| Positive Urgency | 2.57 ± 0.72 | −0.08 |  |
| ^a^Participants excluded from main analyses due to inability to verify US location  ^b^*k*-values reported as log(10) transformed  **t*(63)−3.01, *p*=.004 | | | |
