# Supplementary material for: Changes in substance use, recovery, and quality of life during the initial phase of the COVID-19 pandemic
Source: PLoS One. 2024 May 22;19(5):e0300848. doi: 10.1371/journal.pone.0300848 (PMC11111065; doi:10.1371/journal.pone.0300848)
Supplement: S1 File — (DOCX) [file pone.0300848.s011.docx]

**Supporting Information**

| S1 Table.  Ancillary Data^a^, Demographics | | | | | |  | |  | |  |  |  |  |  |
| --- | --- | --- | --- | --- | --- | --- | --- | --- | --- | --- | --- | --- | --- | --- |
|  | **Active User**  **(*n* = 49)** | | | **Early Recovery**  **(*n* = 64)** | | | | |  |  |  |  |  |  |
|  | *M* ± *SD* |  | *n*(%) | *M* ± *SD* |  | | *n*(%) | |  |  |  |  |  |  |
| Age | 36 ± 11.8 |  | - | 36 ± 9.8 |  | | - | |  |  |  |  |  |  |
| Male | - |  | 38(78) | - |  | | 20(59) | |  |  |  |  |  |  |
| Caucasian | - |  | 39(80) | - |  | | 28(82) | |  |  |  |  |  |  |
| Hispanic/Latino | - |  | 13(27) | - |  | | 13(20) | |  |  |  |  |  |  |
| Bachelor’s Degree | - |  | 20(41) | - |  | | 21(62) | |  |  |  |  |  |  |
| Childhood Household Income | $49,000* |  | - | $49,000* |  | | - | |  |  |  |  |  |  |
| Monthly Disposable Income | $2,000* |  | - | $2,000* |  | | - | |  |  |  |  |  |  |
| Current Student | - |  | 17(35) | - |  | | 23(36) | |  |  |  |  |  |  |
| Daily Smoker (cigarettes) | - |  | 38(78) | - |  | | 59(92) | |  |  |  |  |  |  |
| *Median value  ^a^Participants excluded from main analyses due to inability to verify US location | | | | | | | | | | | | | | |

| **S2 Table.**  **Ancillary Data^a^, Pandemic-related change in use of alcohol in active users** | | | |
| --- | --- | --- | --- |
|  | **Active User (*n* = 49)** | |  |
|  | *M* ± *SD* | (*n*) |  |
| Weekly alcohol consumption | −0.99 ± 6.33 | 49 |  |
| Days spent drinking alcohol | −2.22 ± 10.51 | 49 |  |
| ^a^Participants excluded from main analyses due to inability to verify US location  Means and standard deviations are reported as difference scores (during-COVID−pre-COVID)  Note that illicit substance use was uncommon, with only 9 participants reporting any use pre- or during-COVID (8 out of 9 were cannabis). | | | |

| **S3 Table.**  **Ancillary Data^a^, Illicit substance use by category among active users** | | | | |
| --- | --- | --- | --- | --- |
|  | **Active User (*n* = 49)** | | |  |
|  | **Pre-COVID** | | **During-COVID** |  |
|  | *n*(%) | | *n*(%) |  |
| Cannabis/Marijuana | 6(12) | 6(12) | |  |
| Cocaine/Crack | 3(6) | 2(4) | |  |
| MDMA/Ecstasy | - | 1(2) | |  |
| Amphetamine/Methamphetamine | 1(2) | - | |  |
| Opioid analgesics (including methadone) | - | - | |  |
| Heroin | 1(2) | 1(2) | |  |
| Hallucinogens | 1(2) | - | |  |
| Sedatives/Hypnotics (excluding Benzodiazepines) | - | - | |  |
| Benzodiazepines | - | - | |  |
| Inhalants | - | 1(2) | |  |
| ^a^Participants excluded from main analyses due to inability to verify US location | | | | |

| **S4 Table.**  **Ancillary Data^a^, Resilience and pandemic-related change in alcohol/drug use in active users** | | | |  |
| --- | --- | --- | --- | --- |
|  | **Active User (*n* = 49)** | |  |  |
|  | *r* |  |  |  |
| Weekly alcohol consumption | .20 |  |  |  |
| Days spent consuming alcohol | .09 |  |  |  |
| Number of substances used | .20 |  |  |  |
| Days spent using 1^st^ illicit substance | .25 |  |  |  |
| ^a^Participants excluded from main analyses due to inability to verify US location | | | |  |
|  |  | | | |

| **S5 Table.**  **Ancillary Data^a^, Pandemic-related change in Active User life events and impact ratings** | | |
| --- | --- | --- |
|  | **Active User**  **(*n* = 49)** |  |
|  | *M* ± *SD* |  |
| Number of life events | −0.06 ± 0.66 |  |
| Death of a loved one | −0.41 ± 1.02 |  |
| Divorce/Separation | 0.02 ± 0.72 |  |
| Trouble with the law | 0.02 ± 0.52 |  |
| Personal injury or illness | −0.02 ± 1.22 |  |
| Injury/illness of a loved one | 0.14 ± 1.26 |  |
| Problems with work/school | 0.22 ± 0.87 |  |
| Financial difficulties | 0.00 ± 1.44 |  |
| Loss of employment | −0.10 ± 0.65 |  |
| Increased responsibility | 0.10 ± 1.16 |  |
| Changing/starting work/school | −0.06 ± 0.47 |  |
| Changes in living conditions | 0.06 ± 1.01 |  |
| Victim of crime, violence, or accident | −0.06 ± 0.43 |  |
| ^a^Participants excluded from main analyses due to inability to verify US location  Means and standard deviations are reported as difference scores (during-COVID−pre-COVID) | | |

| **S6 Table.**  **Ancillary Data^a^, Impulsivity and pandemic-related change in alcohol use in active users** | | | | |  |
| --- | --- | --- | --- | --- | --- |
|  | **Active User**  **(*n* = 49)** | **Δ Alcohol Amount** | **Δ Alcohol Frequency** |  |  |
|  | *M* ± *SD* | *r* | *r* |  |  |
| Delay Discounting  *k-*value^b^ | −0.91 ± 1.15 | 0.061 | −0.044 |  |  |
|  |  |  |  |  |  |
| *SUPPS-P Subscales* |  |  |  |  |  |
| Negative Urgency | 2.61 ± 0.62 | −0.174 | −0.146 |  |  |
| Lack of Perseverance | 1.70 ± 0.47 | −0.066 | −0.107 |  |  |
| Lack of Premeditation | 1.81 ± 0.49 | −0.212 | −0.017 |  |  |
| Sensation Seeking | 2.80 ± 0.78 | −0.181 | −0.127 |  |  |
| Positive Urgency | 2.34 ± 0.78 | −0.149 | −0.228 |  |  |
| ^a^Participants excluded from main analyses due to inability to verify US location  ^b^log(10) transformed | | | | | |

| **S7 Table.**  **Ancillary Data^a^, Pandemic-related change in use events and recovery involvement** | | |
| --- | --- | --- |
|  | **Early Recovery**  **(*n* = 64)** |  |
|  | *M* ± *SD* |  |
| Use Events | −0.17 ± 0.46^b^ |  |
| Recovery Group Involvement | −0.27 ± 0.84^c^ |  |
| Sponsor/Mentor in Recovery Group | 0.02 ± 0.60 |  |
| ^a^Participants excluded from main analyses due to inability to verify US location  ^b^*t*(63)= −3.01, *p*=.004  ^c^*t*(63)= −2.53, *p*=.014  Means and standard deviations are reported as difference scores (during-COVID−pre-COVID) | | |

| \| **S8 Table.**  **Ancillary Data^a^, Resilience and pandemic-related change in use and recovery activities** \| \| --- \| | | | |
| --- | --- | --- | --- | --- |
|  | **Early Recovery**  **(*n* = 64)** | |  |
|  | *M* ± *SD* | *r* |  |
| Use events | −0.17 ± 0.05 | −.08 |  |
| Recovery group attendance | −0.27 ± 0.84 | .09 |  |
| Sponsor/mentor in recovery group | 0.02 ± 0.60 | .21 |  |
| ^a^Participants excluded from main analyses due to inability to verify US location  Means and standard deviations are reported as difference scores (during-COVID−pre-COVID) | | | |

| **S9 Table.**  **Ancillary Data^a^, Pandemic-related change in Recovery group life events and impact ratings** | | |
| --- | --- | --- |
|  | **Early Recovery**  **(*n* = 64)** |  |
|  | *M* ± *SD* |  |
| Number of life events | −0.34 ± 1.21 |  |
| Death of a loved one | −0.41 ± 1.35 |  |
| Divorce/Separation | −0.02 ± 0.45 |  |
| Trouble with the law | −0.09 ± 1.00 |  |
| Personal injury or illness | 0.00 ± 1.23 |  |
| Injury/illness of a loved one | −0.11 ± 1.20 |  |
| Problems with work/school | 0.08 ± 1.12 |  |
| Financial difficulties | −0.33 ± 1.08 |  |
| Loss of employment | −0.05 ± 1.13 |  |
| Increased responsibility | −0.14 ± 1.39 |  |
| Changing/starting work/school | −0.20 ± 1.05 |  |
| Changes in living conditions | 0.02 ± 1.24 |  |
| Victim of crime, violence, or accident | 0.08 ± 0.45 |  |
| ^a^Participants excluded from main analyses due to inability to verify US location  Means and standard deviations reported as difference scores (during-COVID−pre-COVID) | | |

| **S10 Table.**  **Ancillary Data^a^, Impulsivity and pandemic-related change in Recovery group use events** | | | |
| --- | --- | --- | --- |
|  | **Early Recovery (*n*=64)** | **Δ Use Events** |  |
|  | *M* ± *SD* | *r* |  |
| Delay Discounting  *k-*value^b^ | −0.76 ± 1.40 | −0.10 |  |
|  |  |  |  |
| *SUPPS-P Subscales* |  |  |  |
| Negative Urgency | 2.57 ± 0.54 | 0.04 |  |
| Lack of Perseverance | 1.83 ± 0.50 | −0.31* |  |
| Lack of Premeditation | 1.74 ± 0.55 | −0.12 |  |
| Sensation Seeking | 2.76 ± 0.57 | 0.04 |  |
| Positive Urgency | 2.57 ± 0.72 | −0.08 |  |
| ^a^Participants excluded from main analyses due to inability to verify US location  ^b^*k*-values reported as log(10) transformed  **t*(63)−3.01, *p*=.004 | | | |
